# Cross-Sectional Comparison of Small Animal [^18^F]-Florbetaben Amyloid-PET between Transgenic AD Mouse Models

**DOI:** 10.1371/journal.pone.0116678

**Published:** 2015-02-23

**Authors:** Matthias Brendel, Anna Jaworska, Eric Grießinger, Christina Rötzer, Steffen Burgold, Franz-Josef Gildehaus, Janette Carlsen, Paul Cumming, Karlheinz Baumann, Christian Haass, Harald Steiner, Peter Bartenstein, Jochen Herms, Axel Rominger

**Affiliations:** 1 Dept. of Nuclear Medicine, University of Munich, Munich, Germany; 2 Dept. of Translational Research I, German Center for Neurodegenerative Diseases (DZNE)—site Munich, University of Munich, Munich, Germany; 3 Laboratory of Neurodegeneration, International Institute of Molecular and Cell Biology, Warsaw, Poland; 4 Department of Nuclear Medicine, University of Erlangen, Erlangen, Germany; 5 Department of Neuroscience and Pharmacology, Copenhagen University, Copenhagen, Denmark; 6 F. Hoffmann-La Roche, Basel, Switzerland; 7 Adolf-Butenandt-Institute, Biochemistry, University of Munich, Munich, Germany; 8 DZNE–German Center for Neurodegenerative Diseases, Munich, Germany; 9 Munich Cluster for Systems Neurology (SyNergy), Munich, Germany

## Abstract

We aimed to compare [^18^F]-florbetaben PET imaging in four transgenic mouse strains modelling Alzheimer’s disease (AD), with the main focus on APPswe/PS2 mice and C57Bl/6 mice serving as controls (WT). A consistent PET protocol (N = 82 PET scans) was used, with cortical standardized uptake value ratio (SUVR) relative to cerebellum as the endpoint. We correlated methoxy-X04 staining of β-amyloid with PET results, and undertook *ex vivo* autoradiography for further validation of a partial volume effect correction (PVEC) of PET data. The SUVR in APPswe/PS2 increased from 0.95±0.04 at five months (N = 5) and 1.04±0.03 (p<0.05) at eight months (N = 7) to 1.07±0.04 (p<0.005) at ten months (N = 6), 1.28±0.06 (p<0.001) at 16 months (N = 6) and 1.39±0.09 (p<0.001) at 19 months (N = 6). SUVR was 0.95±0.03 in WT mice of all ages (N = 22). In APPswe/PS1G384A mice, the SUVR was 0.93/0.98 at five months (N = 2) and 1.11 at 16 months (N = 1). In APPswe/PS1dE9 mice, the SUVR declined from 0.96/0.96 at 12 months (N = 2) to 0.91/0.92 at 24 months (N = 2), due to β-amyloid plaques in cerebellum. PVEC reduced the discrepancy between SUVR-PET and autoradiography from −22% to +2% and increased the differences between young and aged transgenic animals. SUVR and plaque load correlated highly between strains for uncorrected (R = 0.94, p<0.001) and PVE-corrected (R = 0.95, p<0.001) data. We find that APPswe/PS2 mice may be optimal for longitudinal amyloid-PET monitoring in planned interventions studies.

## Introduction

The steadily growing number of patients suffering from Alzheimer’s disease (AD) will place a great burden on healthcare systems in the coming decades, baring development of an effective intervention therapy [1]. Molecular imaging of β-amyloid with positron emission tomography (PET) has given new insight into the progression of AD pathology and has entered clinical diagnostic use [2]. Furthermore, PET imaging is increasingly used for detecting cerebral amyloidosis in transgenic mouse models of AD [3,4]. Small animal PET studies of longitudinal design afford monitoring of the rate of β-amyloid accumulation, and present the possibility of testing interventions for attenuating plaque formation. However, there are some controversies in the literature with regard to the fitness of amyloid-imaging in different AD-mouse models. For example, one PET study failed to reveal any specific signal [5], and another did not detect the known increase of β-amyloid in an aging AD mouse model [6]. Other investigations successfully monitored the progression of amyloidosis in APP23 mice [7] or the early onset of amyloidosis in 5xFAD mice [8]. Comparisons between recent small animal β-amyloid PET studies are difficult, due to the diversity of mouse models, radiotracers, imaging protocols, and quantification strategies [9]. Hence, we wanted to undertake a PET study with an established β-amyloid tracer in several AD mouse strains, applying standardized methods, and correlating PET findings with histological analysis. We therefore aimed to make PET recordings with the [^18^F]-labelled β-amyloid tracer florbetaben [10–12] in four different AD mouse models at a range of ages, with the main focus on APPswe/PS2 mice. Our additional objective was to generalize our validation of partial volume effect correction (PVEC) for quantitation of β-amyloid burden in APPswe mice [13] for APPswe/PS2 mice. Furthermore, we conducted a comprehensive search of the existing literature in order to review systematically the several AD mouse models investigated by florbetaben and other PET tracers for β-amyloid.

## Materials and Methods

### 2.1 Animal models

All experiments were performed in compliance with the National Guidelines for Animal Protection, Germany, with approval of the local animal care committee of the Government of Oberbayern (Regierung Oberbayern), and overseen by a veterinarian. Anaesthesia was performed with isoflurane 1.5%. Euthanasia was performed in deep narcosis by cervical dislocation. Three newly explored and one previously described [3] AD mouse models were assessed by PET at different ages, and ultimately by autoradiography ex vivo and histological methoxy-X04 staining (**Table 1**). Data from the three newly examined models was assessed using groups of mice at different ages, while historical data from APPswe mice had been acquired longitudinally. A total group of 22 age-matched C57Bl/6 mice (WT) served as control material. Details of transgenic strains follow:

**Table 1:** Overview of modalities assessed for the four AD mouse strains.

Mouse Model	Age (mo)	Gender (f/m)	Amyloid PET (N)	Methoxy-X04 Staining (N)	*Ex Vivo* Autoradiography (N)	Amyloid PET uncorrected (SUVR_CTX/CBL_)	Amyloid PET PVE-corrected (SUVR_CTX/CBL_)
PS2APP	5	m	5			0.95 ± 0.04	1.03 ± 0.08
	8	m	7	1	1	1.04 ± 0.03*	1.23 ± 0.06*
	10	m	6			1.07 ± 0.04**	1.38 ± 0.11***
	12	m	2	2	2	1.12 / 1.24	1.47 / 1.55
	16	m	6			1.28 ± 0.06***	1.78 ± 0.16***
	19	m	6	2	2	1.39 ± 0.09***	2.08 ± 0.28***
G384A	5	f	2	2		0.93 / 0.98	1.05 / 1.13
	16	f	1	1		1.11	1.42
APP/PS1dE9	12	m	2	2		0.96 / 0.96	1.05 / 1.08
	24	m	2	2		0.91 / 0.92	0.96 / 1.02
APPswe	10	m	5			0.94 ± 0.03	0.95 ± 0.04
	13	f/m	12	2		0.94 ± 0.04	1.03 ± 0.08
	16	f	8			1.00 ± 0.05*	1.15 ± 0.14**
	20	f	5	8		1.09 ± 0.08***	1.40 ± 0.23***
C57Bl/6	6–22	f	22			0.95 ± 0.03	0.98 ± 0.06

Column 4 indicates the number of animals assessed in PET examinations, whereas columns 5 and 6 indicate the number of hemispheres used for histological and autoradiographic analyses. Uncorrected compared to PVE-corrected SUVR_CTX/CBL_ for all studied groups of mice are provided in columns 7 and 8. mo = months, f = female, m = male. P-values for one-way ANOVA including post-hoc Tukey testing versus youngest littermates are given by:

* p < 0.05;

** p < 0.005;

*** p < 0.001.

Uncorrected SUVR_CTX/CBL_ 95%-CI for pooled C57Bl/6 mice: 0.939–0.962.

**2.1.1 APPswe/PS2 (PS2APP)**. The transgenic B6.PS2APP (line B6.152H) is homozygous for both the human presenilin (PS) 2, N141I mutant and the human APP K670N, M671L mutant. APP and PS 2 transgenes are driven by mouse Thy-1 and mouse prion promoters, respectively. This line had been created by co-injection of both transgenes into C57Bl/6 zygotes, as previously described [14]. Homozygous B6.PS2APP mice were crossed with (C57Bl/6 x DBA/2) x (C57Bl/6 x DBA/2) mice to generate the 152F2 mice, which are hemizygous for both transgenes, and show first plaques in the cortex and hippocampus at 6 months of age [15]. We made PET recordings in 152F2 mice (N = 32), i.e. five animals aged five months, seven animals aged eight months, six animals aged ten months, two aged 12 months, six aged 16 months and six aged 19 months; a subset was used for additional histological analysis. The six ten-month old animals were used for test-retest experiments within an interval of one week.

**2.1.2 APPswe/PS1G384A (G384A)**. APPswexPS1G384A mice were generated by crossing of two pre-existing transgenic mouse lines. APPswe are characterized as stated above, while PS1G384A carries a transgene for mutant PS 1 (G384A mutation; both lines are driven by a Thy-1 promoter). G384A mice (carrying the APP23 transgene) accumulate a significant amount of the 42 amino acid form of ß-amyloid (Aß42) by the time they are two months old; amyloid plaques are at first sparsely distributed, and later on spread from deeper to superficial cortical layers, with highest density in the frontal and somatosensory cortex [16]. We obtained PET recordings in two animals aged five months, and a single animal aged 16 months.

**2.1.3 APPswe/PS1dE9 (APP/PS1dE9)**. APP/PS1dE9 mice [17] overexpress the Swedish mutation of APP, together with PS 1 deleted in exon 9 each driven by the mouse PrP promoter. Overexpression of the transgene construct leads to overproduction of Swedish mutant APP and the PS1 ∆exon9 splice variant with concomitant increase in parenchymal β-amyloid load. These mice develop the first plaques at four months of age mainly in cortical areas and the hippocampus [18]. We obtained PET recordings from two mice aged 12 months and two animals aged 24 months.

**2.1.4 APPswe**. Transgenic APPswe mice overexpressing human amyloid precursor protein (APP) with the Swedish double mutation (K670N, M671L) driven by the mouse Thy1.2 promoter were generated as described earlier [14]. These mice were backcrossed to C57BL/6 mice to generate a line with less than 5% DBA2 background (as determined by microsatellites genotyping). Mice heterozygous for the transgene start to accumulate ß-amyloid at the age of approximately nine months and develop ß-amyloid plaques around the age of 12 months, mainly in the cortical mantle. Historical PET data [3] from 15 APPswe mice imaged at four different ages (10 months: N = 5; 13 months: N = 12; 16 months: N = 8; 20 months: N = 5) was reprocessed using the same standardized parameters as for the newly examined models.

### 2.2 Radiochemistry

The [^18^F]-florbetaben precursor (Piramal Imaging, Berlin) was radiolabeled by the method of Zhang et al. [11], with slight modifications. This procedure yields a radiochemical purity exceeding 98% and specific activity of 50–90 GBq/μmol at the end of synthesis. In brief, [^18^F]fluoride was produced by a cyclotron using the ^18^O(p,n)^18^F reaction and passed through a Chromafix PS-HCO_3_ cartridge (Macherey-Nagel, Düren, Germany) as an aqueous solution in [^18^O]-oxygen enriched water. The cartridge was dried with a stream of helium, and the [^18^F]-activity eluted with 1 ml of Kryptofix 2.2.2 (K_222_)/K_2_CO_3_ solution (12.5 mg of K_222_ and 12.5 μl of 1 M K_2_CO_3_ in CH_3_CN/H_2_O 0.8/0.2 mL). The solvent was removed at 90°C under a helium stream, and the residue was azeotropically dried with 0.8 ml of anhydrous CH_3_CN twice at 90°C, followed by an additional drying *in vacuo* for five min. A solution of mesylate precursor (5 mg) in anhydrous CH_3_CN (1 ml) was added to the reaction vessel containing the dried [^18^F] activity, and heated at 100°C for 12.5 min. Subsequently, HCl (2 M aqueous solution, 0.5 ml) was added and the solution was stirred for another 7.5 min at 100°C. The solution was then cooled to approximately 60°C and a solution containing 1.0 ml of 1 M NaOH and 2.0 ml of 0.1 M ammonium formate was added. The resultant slight cloudy solution was dissolved with 1.0–2.0 ml of CH_3_CN, and purified by HPLC using a Lichrosphere 100 RP18 semi-preperative column (10×250 mm, 10 μm), with mobile phase consisting of CH_3_CN/ammonium formate buffer (0.1 M, with 5 mg/mL sodium ascorbate) 60/40 delivered at a flow rate 5 ml/min. The fraction with the desired product, which eluted with a retention time of 12 min, was collected and diluted with 12 ml water containing 10 mg/ml sodium ascorbate. This solution was passed through a Sep-Pak Light C18 cartridge and rinsed with 10 ml of 20% ethanol in water (also containing 10 mg/ml sodium ascorbate) to separate the hydrolysis by-product from the [^18^F]florbetaben final product, which was then eluted from the cartridge with 1–2 ml ethanol. For mouse experiments, the eluate was divided into 100 μl portions, which were formulated with 900 μl of stabilization solution (1.78 ml PEG400, 6.72 ml H_2_O, 44 mg ascorbic acid, 288 mg sodium ascorbate), followed by adjustment of pH to 7.4 with 75 μl of concentrated phosphate buffer (Braun, Melsungen, Germany). The total synthesis time was 75 min and radiochemical yield was 18% (decay corrected). The product was examined for purity by analytical HPLC using an XDB-C18 analytical column (150×4.6 mm, 5 μm; Agilent Technologies), and mobile phase consisting of CH_3_CN/ammonium formate buffer (0.1 M, with 5 mg/mL sodium ascorbate) 60/40, delivered at a flow rate of 1 ml/min, giving a retention time of 6.2 min, as monitored with serial UV and radiodetection.

### 2.3 Amyloid PET

Image acquisition and reconstruction followed a standardized protocol, with minor modifications from the previously-published methods [3]. Mice were anesthetized with isoflurane (1.5%, delivered via a mask at 3.5 L/min in oxygen) and received bolus injection of 10.2 ± 2.1 MBq of [^18^F]-florbetaben in 150 μL of saline to a tail vein. Following placement in the tomograph (Siemens Inveon DPET), a 15 min transmission scan was obtained using a rotating [^57^Co] point source, followed by a single frame emission recording for the interval 30–60 min p.i.. While still deeply anesthetized, mice were killed by cervical dislocation immediately after the PET scan (62 min p.i.), followed by rapid removal of the brain (68 ± 4 min p.i.). The image reconstruction procedure consisted of a 3-dimensional ordered subset expectation maximization (OSEM) with four iterations and 12 subsets followed by a maximum *a posteriori* (MAP) algorithm with 32 iterations. Scatter and attenuation correction were performed and a decay correction for [^18^F] was applied. With a zoom factor of 1.0 and a 128 x 128 x 159 matrix, a final voxel dimension of 0.78 x 0.78 x 0.80 mm was obtained. PET images were blinded to the reader by coding of the PET files. Manual rigid-body co-registration of emission images to a 3T magnetic resonance imaging (MRI) template, followed by manual rigid-body re-alignment of individual [^18^F]-florbetaben images on a [^18^F]-florbetaben template was accomplished using the PMOD fusion tool (version 3.4; PMOD Technologies Ltd.) [3]. The [^18^F]-florbetaben template had been generated previously by consecutive dual tracer imaging with [^18^F]-FDG and [^18^F]-florbetaben.

For VOI-based analyses, a cerebellar VOI comprising 64 mm³, two bilateral frontal cortex VOIs comprising 15 mm³ each and two bilateral hippocampal VOIs comprising 7mm³ each, were employed for calculation of [^18^F]-florbetaben cortex-to-cerebellum (SUVR_CTX/CBL_) and hippocampus-to-cerebellum standardized uptake value ratios (SUVR_HIP/CBL_). Cortical VOIs were configured larger in comparison to the initial [^18^F]-florbetaben validation study [3] as they proved higher robustness in a recent investigation [13]. The initial oval-shaped VOI was superceded with an anatomically delineated VOI, more accurately accounting for the borders of the neocortex.

Test-retest estimates were acquired in a subset of six PS2APP animals at ten months of age within one week.

### 2.4 Partial volume effect correction

PVEC was first performed in a subset of five PS2APP animals for which *ex vivo* autoradiography was available. After its successful validation, PVEC was applied to the entire dataset. In particular, we used a VOI-based approach implemented in PMOD [19,20] with a full-width-at-half-maximum (FWHM) of 1.72 mm, and a previously-evaluated VOI-mask for ten volumes, consisting of two neocortical VOIs and one cerebellar VOI, along with further VOI comprising the rest of the cerebrum, and an additional six extra-cerebral VOIs (Harderian glands, frontal, superior, basal, spinal, and background). PVEC was additionally performed using a VOI-mask including two hippocampal VOIs instead of the two neocortical VOIs [13]. The neocortical, hippocampal and cerebellar VOIs used for the VOI-mask were identical to those used for calculation of the uncorrected PET data. As described by Rousset et al., regional point spread functions were calculated through integration of single tissue domains’ point spread function, and used for computation of weighting factors representing the contributions of the set of ten tissue domains. Coefficients of a geometric transfer matrix (GTM) were calculated, and PVE-corrected radioactivity concentrations were calculated in the defined VOIs by multiplication of the original PET data by the inverted GTM. The PVE-corrected images thus contained only those regions with VOI-mask definitions, for comparison with VOI-based results for uncorrected PET data. Finally, VOIs were employed for calculation of PVE-corrected [^18^F]-florbetaben cortex-to-cerebellum (SUVR_CTX/CBL_) and hippocampus-to-cerebellum standardized uptake value ratios (SUVR_HIP/CBL_).

### 2.5 *Ex vivo* autoradiography

One hemisphere of five PS2APP animals (8 mo: N = 1; 12 mo: N = 2; 19 mo: N = 2) and of one G384A mouse (16 mo: N = 1) killed at 62 min p.i. (**Table 1**) was frozen by immersion in isopentane (-40°C); 20 μm thick sagittal slices were cut with a Leica CM 1510-1 Cryostat (Leica Microsystems, Nussloch Germany), and mounted on glass slides within 60 minutes post mortem. An imaging plate (Fujifilm; BAS cassette2 2025) was exposed to the slides for ten hours, scanned at 25 μm resolution with Raytest equipment (CR 35 BIO, Dürr Medical, Germany), and analyzed with AIDA image analyzing software (V4.50). Cerebellar and frontal cortical regions-of-interest (ROIs) were drawn analogous to PET-VOIs, and mean radioactivity concentrations per mg tissue equivalent were used for calculation of autoradiographic SUVR_CTX/CBL_ and SUVR_HIP/CBL_ (mean of > 15 slices per animal). As a smaller cortical VOI was used in the initial PET study of APPswe mice [3], we reanalyzed slices from the APPswe mice so as best to match the approach used for the new material. Error-(%) in uncorrected and PVE-corrected *in vivo* PET results was calculated relative to this high resolution “gold standard” of autoradiographic results ex vivo.

### 2.6 Histochemical analyses

Cerebral hemispheres intended for histochemistry were fixed by immersion in 4% parafomaldehyde at 4°C for one week. Several representative 100 μm thick slices in the sagittal plane about 1.5 mm from the midline were cut from each fixed hemisphere using a vibratome (VT 1000 S, Leica, Wetzlar, Germany). The slices were permeabilized overnight at room temperature in 2% Triton X-100 in phosphate buffered saline (PBS) at pH 7.4. Fibrillary ß-amyloid plaques were stained with the fluorescent dye methoxy-X04 (0.01 mg/ml in PBS at pH 7.4 for 15 min) [21]. The unbound dye was removed by three washing steps with PBS, and the slices were then mounted on microscope slides with fluorescent mounting medium (Dako, Germany). 3D image stacks for each hemisphere were acquired on a confocal laser scanning microscope (LSM 780, Jena, Zeiss, Germany). Imaging of the whole slice was performed in tile scan mode which allows automatic stitching of an array of fields of view. For this imaging, the methoxy-X04 was excited at 405 nm, and the emitted light was collected from 410 to 585 nm. The area and number of plaques were automatically counted using Imaris software (Imaris 7.4.2; Bitplane) in a region (0.49 ± 0.06 mm³) defined to match dimensions of the frontal cortex VOI from PET image analysis. Plaque load was calculated as the summed area of all plaques relative to the frontal cortex area. As a smaller cortical VOI was used in the initial PET study of APPswe mice [3], we reanalyzed slices from the APPswe mice so as best to match the approach used for the new material. The histological analyses were performed by an operator blind to the PET results.

### 2.7 Statistics

Group comparisons of uncorrected and PVE-corrected VOI-based PET results measured as SUVR_CTX/CBL_ and SUVR_HIP/CBL_ were performed using one-way ANOVA and the Tukey post-hoc test for multiple comparisons, calculated by IBM SPSS 22 Statistics. Groups of APP-Swe and PS2APP mice with N ≥ 5 were compared against their younger littermates and against the WT group. Comparisons for groups with N < 5 were reported descriptively with their littermates and respective WT animals. For correlation analyses and test-retest analyses, Pearson’s coefficients of correlation (R) were calculated. A threshold of p < 0.05 was considered to be significant for rejection of the null hypothesis.

### 2.8 Literature review

We performed a Pubmed search with the keywords “AD mouse model” and “amyloid PET” aiming to identify all relevant studies. Mouse strain characteristics, the particular radiotracer, and PET image acquisition and reconstruction parameters, as well as summary autoradiographic findings were recorded. Furthermore, Aß42/40 protein levels as well plaque load as functions of mouse age were recorded, when available.

## Results

### 3.1 Amyloid PET

**Figure 1:**
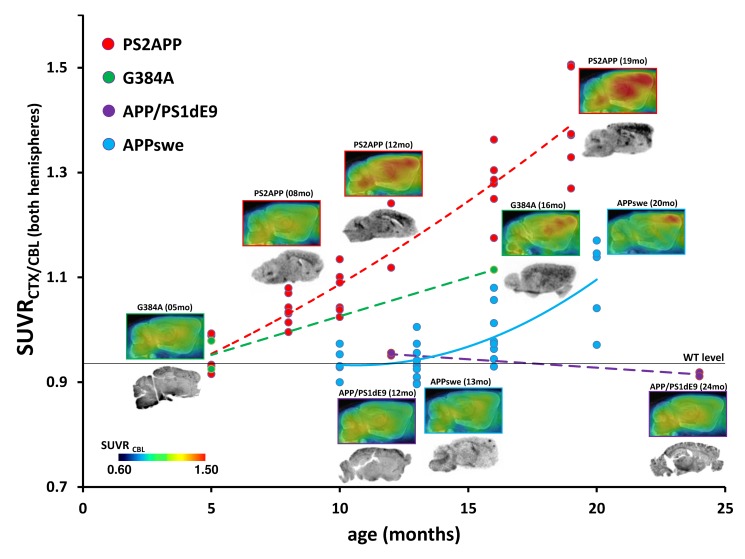
Multi-modal analysis of the four AD mouse strains studies in this cross-sectional [^18^F]-florbetaben PET study. Upper images represent group averaged sagittal PET slices, normalised to the cerebellum and overlayed on an MRI mouse atlas [39]. Dots indicate corresponding assessments of SUVR_CTX/CBL_ in individual mice. Dashed lines express the estimated time dependent progression in PS2APP (red; five months: N = 5; eight months: N = 7; 10 months: N = 6; 12 months: N = 2; 16 months: N = 6, 19 months: N = 6), G384A (green; five months: N = 2; 16 months: N = 1) and APP/PS1dE9 (purple; 12 months: N = 2; 24 months: N = 2) mice, fitted with a polynomial function (for the purposes of illustration). Longitudinal progression in APPswe mice is indicated by a continuous blue line. Lower images depict representative *ex vivo* autoradiography results; autoradiography of APP/PS1dE9 mice and young G384A mice was performed *in vitro*. WT level expresses the mean SUVR_CTX/CBL_ of pooled WT mice (N = 22).

**3.1.1 Cortical findings**. Compared to pooled WT animals (0.95 ± 0.03; 95%-CI: 0.939–0.962; N = 22), PS2APP mice at an age of five months (N = 5) had a similar SUVR_CTX/CBL_ (0.95 ± 0.04). PS2APP animals at eight months (N = 7) had an elevated SUVR_CTX/CBL_ of 1.04 ± 0.03; (+9%; p < 0.05), which further increased to 1.07 ± 0.04 (+12%; p < 0.005) at ten months (N = 6), 1.12/1.24 (+7/19%) at 12 months (N = 2), 1.28 ± 0.06 (+23%; p < 0.001) at 16 months (N = 6) and to 1.39 (+46%; p < 0.001) at 19 months (N = 6), when compared to their five month old littermates. SUVR_CTX/CBL_ results for PS2APP mice age ≥ 8 months differed significantly from WT animals (p < 0.001). G384A mice at five months (N = 2) of age showed [^18^F]-florbetaben SUVR_CTX/CBL_ (0.93/0.98) comparable to that in WT mice; in the single 16 month old animal, SUVR_CTX/CBL_ had seemingly increased to 1.11 (+17%). The SUVR_CTX/CBL_ of two APP/PS1dE9 mice scanned at 12 months of age (0.96/0.96) was 5% lower in two littermates scanned at 24 months of age (0.91/0.92). Standardized re-analysis of the APPswe mice showed an SUVR_CTX/CBL_ of 0.94 ± 0.03 at 10 months of age (N = 5), which remained stable at 13 months (0.94 ± 0.04; N = 12) and then increased to 1.00 ± 0.05 (+6%; p < 0.05) at 16 months (N = 8) and to 1.09 ± 0.08 (+16%; p < 0.001) at 20 months (N = 5), when compared to their baseline (**Fig. 1**).

**3.1.2 Hippocampal findings**. SUVR_HIP/CBL_ was 1.00 ± 0.03 in WT mice; corresponding hippocampal measurements in PS2APP mice aged five, eight or ten months, G384A mice aged five months, APP/PS1dE9 mice aged 12 and 24 months, and APPswe animals at 16 and 20 months were slightly higher than in WT, with SUVR_HIP/CBL_ in the range of 1.04–1.08. Among the PS2APP mice, significantly higher SUVR_HIP/CBL_ values were observed in groups of six mice aged 16 months (1.28 ± 0.07; p < 0.001) and aged 19 months (1.31 ± 0.12; p < 0.001) when compared to their littermates at five months of age. Increased SUVR_HIP/CBL_ was also seen in the single 16 month old G384A mouse (1.13).

### 3.2 Partial volume effect correction and *ex vivo* autoradiography

**Figure 2:**
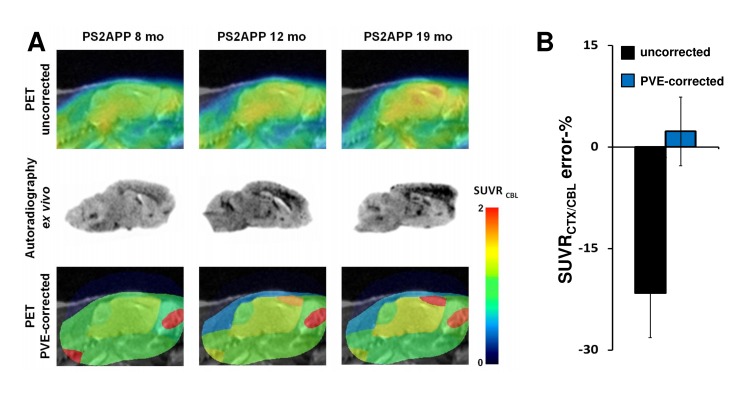
Amyloid-PET and *ex vivo* autoradiography in PS2APP mice before and after PVEC. **(A)** Comparison of uncorrected [^18^F]-florbetaben PET images (upper row), corresponding *ex vivo* autoradiography (mid row) and PVE-corrected PET (lower row) of representative PS2APP mice at 8, 12 and 19 months of age. Sagittal PET images captured 1.6 mm left of the midline were scaled to cerebellum and overlain on a 3T MRI mouse template [13]. PVEC was performed with a 10 region mask (four cerebral and six extracerebral VOIs). **(B)** Error-(%) (±SD) of uncorrected (black bar) and PVE-corrected (blue bar) data versus *ex vivo* autoradiography are shown for the whole group of PS2APP mice.

Applying the PVEC increased the SUVR_CTX/CBL_ measurements by 30 ± 11% (range: 12–53%) in the five histologically analyzed PS2APP animals, with the more pronounced effect seen in those animals with higher uncorrected SUVR_CTX/CBL_. Values of SUV in the cerebellum VOI increased by 8 ± 4% with PVEC, propagating to a nonlinear relationship between PVE-corrected and uncorrected SUVR_CTX/CBL,_ which was described by y = 1.16x^1.65^. Corrected SUVR_CTX/CBL_ was 1.18 in the single PS2APP mouse aged eight months and age-dependently increased to 1.47/1.55 (+25/31%) in the two mice aged 12 months and to 1.68/2.04 (+42/73%) in two mice aged 19 months (**Fig. 2A**). Applying PVEC with the hippocampal VOI-mask increased SUVR_HIP/CBL_ by 20 ± 5%, in a nonlinear relationship described by y = 1.09x^1.34^.

SUVR_CTX/CBL_ by autoradiography *ex vivo* was 13–30% higher in PS2APP animals (1.18 at eight months, 1.36/1.53 at 12 months, and 1.60/2.05 at 19 months) relative to PET results without PVEC; this difference indicates an overall mean 22 ± 7% underestimation of amyloid burden by PET. PVEC reduced this mean bias to a non-significant overestimation by 2 ± 5% (**Fig. 2B**).

Autoradiographic SUVR_HIP/CBL_ was 1.11 in an eight month old PS2APP animal, and increased to 1.28/1.38 in two littermates aged 12 months and 1.27/1.55 in the pair aged 19 months. Uncorrected PET data showed a bias of −5 ± 4% versus +14 ± 12% with PVEC, indicating increased bias and variance. SUVR_CTX/CBL_ by autoradiography *ex vivo* in the single G384A mouse was 1.41, i.e. only 3% higher than the PVE-corrected PET SUVR_CTX/CBL_ of 1.37, and 29% higher than the uncorrected PET SUVR_CTX/CBL_ of 1.09 in the right hemisphere.

### 3.3 PVEC application to the entire dataset

**Figure 3:**
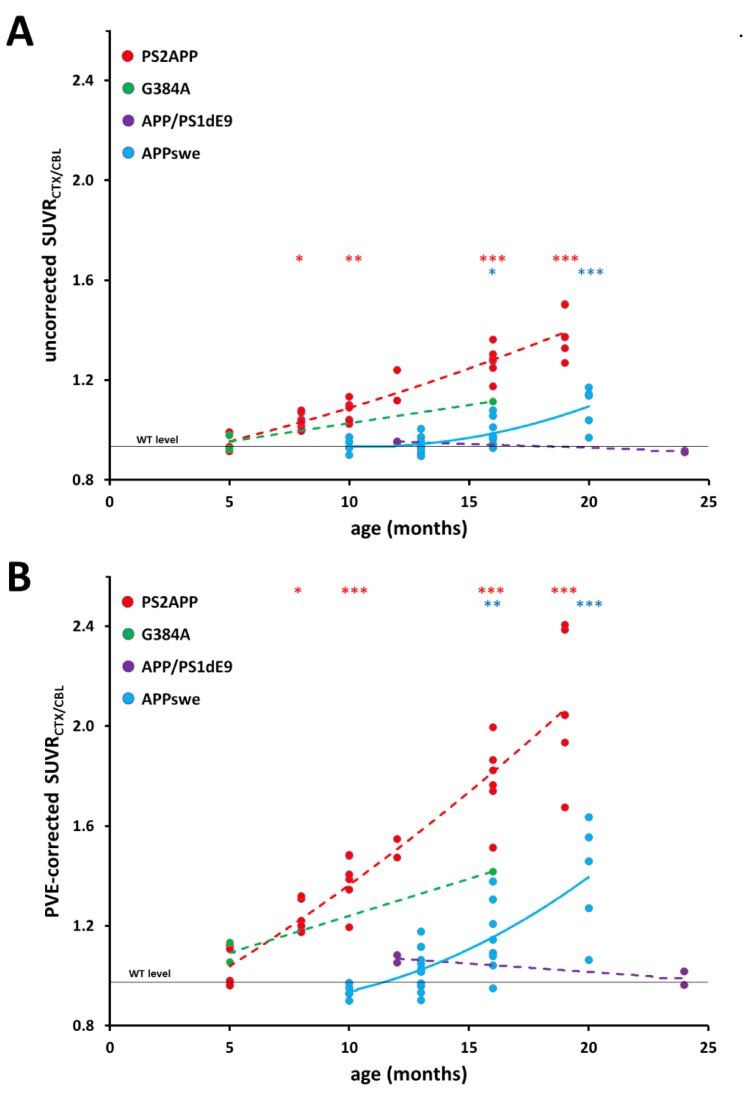
Comparison of uncorrected (A) and PVE-corrected (B) SUVR_CTX/CBL_ of the entire dataset. Dots indicate corresponding assessments of SUVR_CTX/CBL_ in individual mice. Dashed lines express the estimated time dependent progression in PS2APP (red; five months: N = 5; eight months: N = 7; 10 months: N = 6; 12 months: N = 2; 16 months: N = 6, 19 months: N = 6), G384A (green; five months: N = 2; 16 months: N = 1) and APP/PS1dE9 (purple; 12 months: N = 2; 24 months: N = 2) mice, fitted with a polynomial function (for the purposes of illustration). Longitudinal progression in APPswe mice is indicated by a continuous blue line. P-values for one-way ANOVA (incl. post hoc Tukey) testing of PS2APP and APPswe mice versus youngest littermates were as indicated: * p < 0.05; ** p < 0.005; *** p < 0.001.

Uncorrected and PVE-corrected SUVR_CTX/CBL_ of all mice are provided in **Table 1**. PVE-corrected values distinctly increased the estimated slope, especially in the PS2APP strain and the difference between young and old PS2APP animals (**Fig. 3**).

### 3.4 Test-retest

Uncorrected SUVR_CTX/CBL_ was 1.07 ± 0.04 (range: 1.02–1.13) for the ten month old PS2APP animals (N = 6) in the first PET session and 1.07 ± 0.03 (range: 1.04–1.12) when rescanned one week later. PVE-corrected SUVR_CTX/CBL_ results were 1.38 ± 0.11 (range: 1.20–1.48) in the first scan and 1.39 ± 0.09 (range: 1.23–1.51) in the rescan. Correlation analysis between individual test and re-test results gave R = 0.90 (p < 0.05) for uncorrected and R = 0.94 (p < 0.005) for PVE-corrected data.

### 3.5 Histochemical analyses

**3.5.1 Plaque analysis**. PS2APP mice had close to 1300 mainly compact plaques in the frontal cortex at all ages studied, but the average size and maximum plaque diameter increased with, leading to a plaque load of up to 12.2% in 19 month old animals; plaques were also abundant in olfactory bulb and thalamus. G384A mice showed a fourfold increase in number of plaques from 5 to 16 months of age, with a concomitant increase in mean diameter. APP/PS1dE9 mice revealed a sparse distribution of small plaques in cerebral cortex, and also in cerebellum. Re-analysis of material from APPswe mice aged 20 months showed distinctly larger plaques (max. diameter 243 μm) compared to the three other strains (all < 100 μm). Plaque load in 20 month old APPswe mice (6.3 ± 3.1%) was comparable to that of the 16 month old G384A mouse (7.6%). For details see **Table 2**.

**Table 2:** Comparison of histological results obtained from the four different AD mouse strains (columns 1–3).

Mouse Model	Age (mo)	N	Plaque Load (PL-%)	Plaque Count (N)	Plaque Density (N/mm^3^)
PS2APP	8	1	4.9	1408	2930
PS2APP	12	2	8.5 / 10.2	1496 / 1402	4121 / 3762
PS2APP	19	2	9.6 / 12.2	1281 / 1460	2982 / 2936
G384A	5.5	2	0.8 / 2.2	209 / 477	346 / 852
G384A	16	1	7.6	1216	2866
APP/PS1dE9	12	2	1.8 / 2.0	599 / 522	1256 / 1185
APP/PS1dE9	24	2	2.0 / 4.2	361 / 839	731 / 1862
APPswe	20	5	6.3 ± 3.1	349 ± 105	715 ± 244

Plaque load percentage (PL-%) and plaque numbers in the frontal cortex VOI are reported (columns 4 and 5), as well as the density of plaques (column 6). Exemplified sagittal slices from methoxy-X04 staining of ß-amyloid plaques are shown together with illustrations of the plaque size distribution in **S1 Table**.

**Figure 4:**
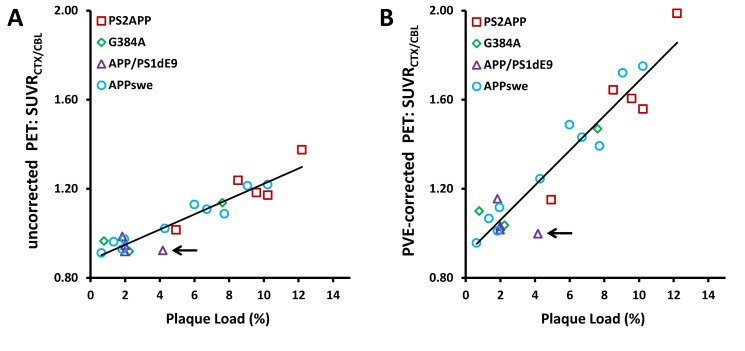
Cross-sectional correlation of plaque load percentage (methoxy-X04 staining) with uncorrected (R = 0.94; p < 0.001) (A) and PVE-corrected (R = 0.95; p < 0.001) (B) [^18^F]-florbetaben PET results (SUVR_CTX/CBL_) for PS2APP (red squares; eight months: N = 1; 12 months: N = 2; 19 months: N = 2), G384A (green diamonds; five months: N = 2; 16 months: N = 1), APP/PS1dE9 (purple triangle; 12 months: N = 2; 24 months: N = 2) and APPswe (blue circles; 13 months: N = 2; 20 months: N = 8) mice. A total of 22 hemispheres from 19 animals were examined by histology. Black arrow depicts an APP/PS1dE9 mouse with extensive cerebellar plaque load, which resulted in an anomalously low PET SUVR_CTX/CBL_.

**3.5.2 Cross-sectional plaque load correlation**. We found a very high correlation between cortical plaque load in all 22 hemispheres stained with methoxy-X04 and the uncorrected SUVR_CTX/CBL_ (R = 0.94, p < 0.001; **Fig. 4A**) as well as with the PVE-corrected SUVR_CTX/CBL_ (R = 0.95, p < 0.001; **Fig. 4B**) as assessed by [^18^F]-florbetaben PET. The only outlier was a single 24 month old APP/PS1dE9 mouse (arrow), in which too low SUVR_CTX/CBL_ was apparently due to bias in PET results arising from plaques in the cerebellum reference region.

## Discussion and Review of the Literature

We present the first cross-sectional small animal PET study with a single [^18^F]-labelled radiotracer for β-amyloid under uniform standardized experimental settings in several established AD mouse models. We found distinct differences in time course and anatomic pattern of β-amyloid deposition among the four AD mouse strains investigated. PET findings of cortical [^18^F]-florbetaben binding in AD mouse models matched precisely the distribution of plaques verified by histology with methoxy-X04 staining. Of the four AD mouse strains examined, PS2APP mice were judged most suitable for future intervention studies, based on the presence of [^18^F]-florbetaben binding in mice aged only 8 months, continued substantial β-amyloid accumulation to at least 19 months, and lack of specific binding in the cerebellum reference region.

### 4.1 PS2APP animals

A previous study with [^18^F]-florbetapir in double transgenic APP/PS1 mice showed an increasing PET signal from three to five months, but no further progression to 12 months [6]; the transgenic animals attained a constant SUVR_CTX/CBL_ for [^18^F]-florbetapir of only 1.1. This PET finding was in contrast to histopathological plaque load, which increased 3-fold from four to eight months of age, and further doubled by 19 months of age [22]. In contrast, our PS2APP mice showed 8% elevated [^18^F]-florbetaben SUVR_CTX/CBL_ at eight months relative to WT mice, and then a continuous progression of cortical binding to 19 months. The PET findings are in line with histology, and with earlier reports of β-amyloid protein levels in the PS2APP strain [15]. Compared to the human pathology compact plaques of different sizes represent the majority in old PS2APP mice, whereas diffuse and cored plaques are predominating in AD patients [23]. Similarly there was general agreement between PET signal from [^11^C]-Pittsburgh Compound B ([^11^C]-PiB), plaque load and Aß42 protein in homozygous ARTE 10 mice, which were evaluated at ages up to 21 months [4]. A recent report of 5xFAD mice examined with [^11^C]-PiB and [^18^F]-florbetapir at 11 months of age showed distinctly elevated cortical uptake of both tracers (+20%) compared to WT mice [8]. Thus, the report of a plateau of [^18^F]-florbetapir uptake in aging APP/PS1 mice may be anomalous.

### 4.2 G384A animals

Double transgenic mice carrying mutations of APP and the G384A mutation are reported to develop solid amyloid plaques as early as two months of age, when the APP23 strain was used for the cross-breeding [16]. In our study methoxy-X04 staining revealed solid plaques at a low plaque load of 1.5% in G384A mice aged five months including the APPswe mutation. However we did not see consistently elevated [^18^F]-florbetaben PET signal in these animals, but only in a single mouse aged 16 months which presented a 5-fold higher plaque load compared to five months. We concede that we examined relatively few G384A mice, i.e. two animals at 5 months and a single animal aged 16 months. As the extent of amyloidosis in transgenic AD models can be highly variable, the seeming increase in the single G384A mouse has to be interpreted with caution. Nonetheless, the evidence for a positive ß-amyloid PET signal was unambiguous in the aged animal of this strain, as its signal clearly exceeded the upper bound of the 95%-CI in WT mice.

There is abundant precedent in the literature for discrepancies between age of plaque onset and detectability with small animal amyloid PET. Thus, several explorations of APP23 mice imaged with [^11^C]-PiB [7,24,25] showed that transgenic mice with only an APP mutation can indeed serve for β-amyloid imaging. Sparse plaque formation was evident histologically in APP23 mice as early as six months, and aged animals exhibited numerous large dense-core plaques, high Aß40 level and a remarkable plaque load (**Table 3**). Nonetheless, elevated [^11^C]-PiB PET signal was first seen at 15 months of age. Similarly, we have reported low [^18^F]-florbetaben SUVR_CTX/CBL_ in APPswe animals aged ten months, even though histological plaque formation is already established at nine months. Indeed, our 13 month old mouse with histologically detectable plaques did not show a discernibly elevated PET signal. We have earlier calculated, based upon the concordance of PET signal and histology that the [^18^F]-florbetaben PET method was sensitive for a 1.5% plaque load in APPswe mice [3]. This sensitivity limit, which likely accounts for the temporal gap between onset of pathology and detection threshold by PET, must be determined for each strain in order to anticipate the statistical power of treatment studies, especially for AD models without acceleration of amyloidosis due to a presenilin mutation.

**Table 3:** Comprehensive overview of histological studies in ten AD mouse strains which have been investigated in PET studies to date, including the present work.

Age	Tg2576 [22,25,28–31]		APP23 [22,25,32–35]		APP23xPS1G384A [16,32]		APPswe female [14]		PS2APP male [15]		APP/PS1dE9 [17,26,27]		APP/PS1-21 [22]		ARTE10 tg [4,36]		ARTE10 tg-tg [4,36]		5xFAD [31]	
(mo)	Aß42 (Aß42/Aß40)	PL-%	Aß42 (Aß42/Aß40)	PL-%	Aß42 (Aß42/Aß40)	PL-%	Aß42 (Aß42/Aß40)	PL-%	Aß42 (Aß42/Aß40)	PL-%	Aß42 (Aß42/Aß40)	PL-%	Aß42 (Aß42/Aß40)	PL-%	Aß42 (Aß42/Aß40)	PL-%	Aß42 (Aß42/Aß40)	PL-%	Aß42 (Aß42/Aß40)	PL-%
1–2					0.5 (2.1)	0.7							0.8 (4.8)	0.9					70.1 (3.9)	
3–4	0.1 (0.3)								2.2 (54.2)		0 (0.0)		11.1 (3.4)	3.4					320.2 (2.5)	
5–6	0 (0.2)		0 (0.1)						43.3 (5.8)		0.4 (5.2)								860.4 (2.6)	
7–8	0.1 (0.4)			0.3							3.8 (2.2)		67.0 (9.9)	9.9						
9–10	0.1 (0.1)				117.3 (0.7)	9.1			47.1 (7.3)		2.2 (4.0)	3.5					179.0 (1.0)	4.7	1090.0 (2.8)	
11–12	0.8 (0.7)	0.1					0.1 (0.2)		107.8 (7.5)		4.6 (2.5)					2.1		9.2	1200.0 (2.9)	
13–14								0.4												
15–16	1.1 (0.1)				280.3 (2.3)															
17–18		3.5					7.2 (0.1)		76.1 (4.9)											
19–20								4.1			19.2 (1.6)			20.9		10.5		35.2		
21–22	11.6 (0.2)																572.0 (0.9)	11.8		
23–24	21.3 (0.2)	6.1	42.7 (0.1)				49.2 (0.2)		171.9 (5.3)						145.0 (0.9)	7.7				
25–26			135.3 (0.2)	24.1																

Protein levels of ß-amyloid of 42 amino acid length (Aß42), ratio of ß-amyloid 42 to 40 (Aß42/40) and cortical plaque load percentage (PL-%) were compared for mice of different ages (column 1). Protein levels are given in ng protein/mg wet brain weight. N.b; calculations of PL-% were obtained using various techniques, and are therefore only approximately comparable between studies. Conversion factors used to aid this comparison were: Aß40: 4330 g/mol; Aß42: 4514 g/mol; total protein (g) per wet brain (g) = 9.4/100.

### 4.3 APP/PS1dE9 animals

From our data in four mice, the APP/PS1dE9 model appeared unsuitable for PET quantitation of relative cerebral amyloid burden due to the presence of ß-amyloid pathology in the reference region (cerebellum). Furthermore, cortical plaque levels in even the most aged mice (24 mo) only slightly exceeded our detection threshold for [^18^F]-florbetaben PET (noted above), with the caveat that one must take into the small sample size. In concordance with this finding *in vivo*, the plaque load (**Table 2**) and Aß42 protein levels (**Table 3**) in the APP/PS1dE9 strain appear far lower than those of APP23, G384A and PS2APP mice, which likely accounts for the lower [^18^F]-florbetaben PET signal in 24 month old APP/PS1dE9 mice when compared to other mouse strains at this age. Earlier reports on APP/PS1dE9 mice did not report the presence of cerebellar plaques, albeit these studies were confined to younger animals [17,26], or only investigated forebrain for histological analyses [27]. However, a very recent longitudinal [^11^C]-PiB PET study of APP/PS1dE9 mice reported cerebellar plaques in 19 month old mice [7]. In our hands, aged APP/PS1dE9 mice had comparable ß-amyloid levels in cerebellum and in cortical areas, such that the SUVR_CTX/CBL_ actually declined from 12 to 24 months of age, ultimately falling below the lower bound of the 95%-CI in WT mice. The cerebellum has been used as a reference region in nearly all recent small animal β-amyloid PET studies, although its histological suitability was previously established only for ARTE 10 and APPswe mice [3,4]. The plaque-free pons might be proposed as an alternate reference region, but due to its small size in mice, we expect that its use would bring considerable penalty in precision of binding ratios. Furthermore, plaques in APP/PS1dE9 mice had 3-fold higher plaque area in a 3D6-immunohistochemistry study which also detects diffuse β-amyloid deposits, in contrast to thioflavin S staining, which is only sensitive to dense aggregates [26]. Our approach of using a radiotracer and a histological marker with similar binding characteristics gave more concordant results, in that low plaque load matched with low *in vivo* [^18^F]-florbetaben binding. Therefore, we agree with Snellman et al. (2013) that low SUVR_CTX/CBL_ magnitude likewise reflected inherently lower radiotracer binding in 24 month old APP/PS1dE9 mice when compared to other AD mouse strains at this age.

### 4.4 Partial volume effect correction

A previous report on [^18^F]-florbetapir PET in APP/PS1 mice reported 20–30% higher SUVR_CTX/CBL_ in autoradiographic results *ex vivo* compared to PET findings [6]. We likewise found nearly identical discrepancies by method in young and aged PS2APP animals, as well as the re-evaluated APPswe mice, which we attribute to errors in regional quantitation arising from uncorrected PVEs. Application of the Rousset method for cross-talk correction essentially removed the bias in our PET estimates [13]. The present generalization of the PVEC algorithm to another mouse model helps establish the validity of the approach to improved quantitation; there have been but few investigations of PVEC in the rodent brain. We find that cortical spill-over is otherwise a significant factor in underestimating β-amyloid signal, especially in mice with high plaque load. The systematic use of PVEC will help to reduce quantification bias in future studies, and should improve sensitivity of PET for detecting effects of interventions. Unlike in neocortex, uncorrected PET results in hippocampus were more congruent with the autoradiographic results, no doubt due to the VOI being surrounded by tissues with similar radioactivity concentrations, resulting in lesser net spill-out of signal. Indeed, PVEC for hippocampus resulted in overcorrection and excessive variance, which is unsurprising for such small structures (6.6 mm³). Despite the limitations of PVEC for the mouse hippocampus, *in vivo* β-amyloid imaging of that structure may yet be possible without correction, as suggested by our finding of increasing SUVR_HIP/CBL_ in aged PS2APP animals.

### 4.5 Cross-sectional plaque load correlation

The reason for the general finding of low binding of β-amyloid PET radiotracers in the brains of young transgenic mouse models of AD, or even absence of specific signal in some strains, despite abundant β-amyloid levels, is a matter of considerable controversy. Maeda et al. [25] even reported lower *in vitro* binding of [^11^C]-PiB to plaques in double transgenic PS-1/APP mice aged eight months compared to Tg2576 animals aged 23 months with similar Aß42 levels; they concluded that plaque formation in accelerated models impart fewer radiotracer binding sites due to post-translation factors resulting in lower AßN3-pyroglutamate composition. We found an excellent cross-sectional correlation of PET results and methoxy-X04 staining for our four AD models (**Fig. 4**). This probably reflects the similar binding/staining properties of [^18^F]-florbetaben and methoxy-X04, as both detect fibrillar but not diffuse β-amyloid depositions [10,21]. Although different mouse strains in this investigation had a high variability of plaque sizes, numbers and morphology, the relationship between stained fibrillar β-amyloid intensity and radiotracer binding was nearly linear. Of note is that in cortex of AD patients the compact plaque type occurs at only 10%, with cored and diffuse plaques dominating. Furthermore all three plaque types differ with respect to Aβ species, with diffuse and compact plaques containing mostly Aβ42, while cored plaques contain Aβ40 [23].

We conclude that histological analyses with this Congo red derivate are indeed predictive of [^18^F]-florbetaben binding sites to be imaged by PET, with the caveat that the *in vivo* method is insensitive to low plaque loads (<1.5%). This is probably related to factors such as low specific binding signal, the limited spatial resolution of PET, and image noise. Additionally, the impact of different specific activities of [^18^F]-florbetaben have not yet been tested systematically in preclinical settings, and might conceivably have influenced the detectability threshold in our study [25], however in a preliminary analysis in a series of PS2APP mice no conspicuous effect on scan results as a function of time from end of synthesis could be observed (data not shown).

Comparisons between β-amyloid PET studies in AD mouse models must be made with some caution, due to diverse methodological differences, including the particular radiotracers, emission recording time-frames and reconstruction parameters. Cortex-to-reference region estimates for WT animals fall within a narrow range in a number of recent small animal PET studies using different radiotracers irrespective of age (**Table 4**), which predicts general stability of the method. On the other hand, PET signals in transgenic animals are more difficult to compare, as sizes and anatomic compositions of target VOIs, i.e. forebrain, entire cortex, or hot spot, greatly influence PET quantification. In published studies, APP23, ARTE 10, APPswe, PS2APP, and G384A mice have all been found to meet criteria for PET monitored treatment studies, whereas Tg2576, APP/PS1-21 and APP/PS1dE9 animals lacked sufficient or longitudinal increases of the PET signal. Limited evidence also supports the use of 5xFAD mice for longitudinal studies.

**Table 4:** Comprehensive overview of small animal β-amyloid PET studies in transgenic AD mice.

Mouse Model / Study	Age (mo)	N	Radiotracer	Activity (MBq)	Scanner	Reconstr.	SC	AT	M	Voxel size (mm^3^)	EM time (min p.i.)	PET: SUVR_CTX/CBL_	PET: cortical BP_ND_	Corresp. PET of WT SUVR or BP_ND_	*Ex vivo* Autorad. SUVR_CTX/CBL_
Tg2576 [37]	22	6	[^11^C]-PiB	13–46	NIH	3D OSEM	x	x	x	0.6 x 0.6 x 1.1	12–30	1.06 ± 0.04		0.98 ± 0.07	
Tg2576 [5]	14	6	[^18^F]-FDDNP	4–10	F220	FORE, 2D FBP	x	o	x	n.r.	0–60	0.92 ± 0.10		1.00 ± 0.09	
Tg2576 [7]	9	3	[^11^C]-PiB	8.7 ± 1.4	Inveon	FORE, 2D FBP	o	o	x	0.8 x 0.8 x 0.8	5–60		0.97 ± 0.06**		
	12	2	[^11^C]-PiB	8.7 ± 1.4	Inveon	FORE, 2D FBP	o	o	x	0.8 x 0.8 x 0.8	5–60		0.95 ± 0.04**		
	19	2	[^11^C]-PiB	8.7 ± 1.4	Inveon	FORE, 2D FBP	o	o	x	0.8 x 0.8 x 0.8	5–60		1.06 ± 0.04**		
	22	2	[^11^C]-PiB	8.7 ± 1.4	Inveon	FORE, 2D FBP	o	o	x	0.8 x 0.8 x 0.8	5–60		1.02 ± 0.08**	1.04 ± 0.01**	
APP23 [25]	21.4	11	[^11^C]-PiB	30 ± 6.8	F220	FORE, 2D FBP	x	o	x	n.r.	0–60		0.30 ± 0.04	0.00 ± 0.01	3.2 ± 0.6
	17	5	[^11^C]-PiB	31 ± 6.8	F220	FORE, 2D FBP	x	o	x	n.r.	0–60		0.06		
	22	5	[^11^C]-PiB	30 ± 6.8	F220	FORE, 2D FBP	x	o	x	n.r.	0–60		0.36		
	27	5	[^11^C]-PiB	31 ± 6.8	F220	FORE, 2D FBP	x	o	x	n.r.	0–60		0.64		
APP23 [24]	22.6	12	[^11^C]-PiB	30.3 ± 5.5	F220	FORE, 2D FBP	x	o	x	n.r.	0–60		0.31 ± 0.05		
APP23 [7]	7	2	[^11^C]-PiB	8.7 ± 1.4	Inveon	FORE, 2D FBP	o	o	x	0.8 x 0.8 x 0.8	5–60		1.02 ± 0.07**		
	12	2	[^11^C]-PiB	8.7 ± 1.4	Inveon	FORE, 2D FBP	o	o	x	0.8 x 0.8 x 0.8	5–60		0.98 ± 0.07**		
	15	1	[^11^C]-PiB	8.7 ± 1.4	Inveon	FORE, 2D FBP	o	o	x	0.8 x 0.8 x 0.8	5–60		1.19**		
	18	3	[^11^C]-PiB	8.7 ± 1.4	Inveon	FORE, 2D FBP	o	o	x	0.8 x 0.8 x 0.8	5–60		1.38 ± 0.03**		
	21	1	[^11^C]-PiB	8.7 ± 1.4	Inveon	FORE, 2D FBP	o	o	x	0.8 x 0.8 x 0.8	5–60		1.34**	1.08 ± 0.02**	
APP/PS1-21 [6]	3	5	[^18^F]-Florbetapir	10–15	Inveon	FORE, 2D OSEM	x	o	x	0.4 x 0.4 x 0.8	30–60	1.01		1.00	1.30
	5	5	[^18^F]-Florbetapir	10–15	Inveon	FORE, 2D OSEM	x	o	x	0.4 x 0.4 x 0.8	30–60	1.11		1.01	
	8	5	[^18^F]-Florbetapir	10–15	Inveon	FORE, 2D OSEM	x	o	x	0.4 x 0.4 x 0.8	30–60	1.06		0.97	1.61
	12	5	[^18^F]-Florbetapir	10–15	Inveon	FORE, 2D OSEM	x	o	x	0.4 x 0.4 x 0.8	30–60	1.09		0.96	1.65
ARTE 10 (tg) [38]	24.6	18	[^11^C]-PiB	20.2 ± 5.6	F120	FORE, 2D FBP	x	o	o	0.8 x 0.8 x 0.8	36–45	1.26 ± 0.15*			
ARTE 10 (tg) [4]	23.2	5	[^11^C]-PiB	13.9	F120	FORE, 2D FBP	x	o	o	0.9 x 0.9 x 0.8	0–60		0.28 ± 0.06	−0.10 ± 0.03	1.90 ± 0.26
(tg-tg)	9.2	7	[^11^C]-PiB	45.2	F120	FORE, 2D FBP	x	o	o	0.9 x 0.9 x 0.8	0–60		0.12 ± 0.03	−0.10 ± 0.03	1.25 ± 0.07
(tg-tg)	21.1	4	[^11^C]-PiB	22.4	F120	FORE, 2D FBP	x	o	o	0.9 x 0.9 x 0.8	0–60		0.51 ± 0.13	−0.10 ± 0.03	2.54 ± 0.27
APP/PS1dE9 [7]	9	2	[^11^C]-PiB	8.7 ± 1.4	Inveon	FORE, 2D FBP	o	o	x	0.8 x 0.8 x 0.8	5–60		1.03 ± 0.06**		
	12	2	[^11^C]-PiB	8.7 ± 1.4	Inveon	FORE, 2D FBP	o	o	x	0.8 x 0.8 x 0.8	5–60		1.07 ± 0.04**		
	15	2	[^11^C]-PiB	8.7 ± 1.4	Inveon	FORE, 2D FBP	o	o	x	0.8 x 0.8 x 0.8	5–60		± 0.04**		
	19	2	[^11^C]-PiB	8.7 ± 1.4	Inveon	FORE, 2D FBP	o	o	x	0.8 x 0.8 x 0.8	5–60		0.98 ± 0.03**	1.01 ± 0.02**	
APP/PS1dE9 (this paper)	12	2	[^18^F]-Florbetaben	10.2 ± 2.1	Inveon	3D OSEM, MAP	o	o	x	0.8 x 0.8 x 0.8	30–60	0.96 / 0.96		0.96 ± 0.02	
	24	2	[^18^F]-Florbetaben	10.2 ± 2.1	Inveon	3D OSEM, MAP	o	o	x	0.8 x 0.8 x 0.8	30–60	0.91 / 0.92		0.96 ± 0.03	
5xFAD [8]	11.2	10	[^18^F]-Florbetapir	9.3 ± 3.4	R4	n.r.	x	x	x	n.r.	45–75	1.19 ± 0.02*		1.03 ± 0.02*	
	10.5	10	[^11^C]-PiB	11.5 ± 3.6	R4	n.r.	x	x	x	n.r.	35–65	1.21 ± 0.04*		1.00 ± 0.03*	
G384A (this paper)	5.5	2	[^18^F]-Florbetaben	10.2 ± 2.1	Inveon	3D OSEM, MAP	o	o	x	0.8 x 0.8 x 0.8	30–60	0.93 / 0.98		0.99 ± 0.03	
	16	1	[^18^F]-Florbetaben	10.2 ± 2.1	Inveon	3D OSEM, MAP	o	o	x	0.8 x 0.8 x 0.8	30–60	1.11		0.96 ± 0.02	1.41
APPswe [3]	10	5	[^18^F]-Florbetaben	8.9 ± 2.7	Inveon	3D OSEM, MAP	o	o	x	0.8 x 0.8 x 0.8	30–60	0.94 ± 0.03		0.96 ± 0.02	
	13	10	[^18^F]-Florbetaben	8.9 ± 2.7	Inveon	3D OSEM, MAP	o	o	x	0.8 x 0.8 x 0.8	30–60	0.94 ± 0.04		0.95 ± 0.02	1.01 ± 0.04
	16	8	[^18^F]-Florbetaben	8.9 ± 2.7	Inveon	3D OSEM, MAP	o	o	x	0.8 x 0.8 x 0.8	30–60	1.00 ± 0.05		0.96 ± 0.02	
	20	5	[^18^F]-Florbetaben	8.9 ± 2.7	Inveon	3D OSEM, MAP	o	o	x	0.8 x 0.8 x 0.8	30–60	1.09 ± 0.08		0.96 ± 0.01	1.48 ± 0.19
PS2APP (this paper)	5	5	[^18^F]-Florbetaben	10.8 ± 1.7	Inveon	3D OSEM, MAP	o	o	x	0.8 x 0.8 x 0.8	30–60	0.95 ± 0.04		0.99 ± 0.03	
	8	7	[^18^F]-Florbetaben	10.5 ± 1.6	Inveon	3D OSEM, MAP	o	o	x	0.8 x 0.8 x 0.8	30–60	1.04 ± 0.03		0.98 ± 0.03	1.18
	10	6	[^18^F]-Florbetaben	11.4 ± 1.3	Inveon	3D OSEM, MAP	o	o	x	0.8 x 0.8 x 0.8	30–60	1.07 ± 0.04		0.96 ± 0.03	
	12	2	[^18^F]-Florbetaben	10.2 ± 2.1	Inveon	3D OSEM, MAP	o	o	x	0.8 x 0.8 x 0.8	30–60	1.12 / 1.24		0.96 ± 0.02	1.36 / 1.53
	16	6	[^18^F]-Florbetaben	10.8 ± 2.5	Inveon	3D OSEM, MAP	o	o	x	0.8 x 0.8 x 0.8	30–60	1.28 ± 0.06		0.97 ± 0.03	
	19	6	[^18^F]-Florbetaben	10.2 ± 2.1	Inveon	3D OSEM, MAP	o	o	x	0.8 x 0.8 x 0.8	30–60	1.39 ± 0.09		0.95 ± 0.04	1.60 / 2.05

Results of eleven published studies using eight different strains at multiple ages (columns 1–3) were compared for particular tracer (columns 4–5), PET instrumentation (column 6) acquisition and reconstruction parameters (columns 7–12), PET results (columns 13–15) and autoradiographic results *ex vivo* (column 16).

* indicates whole forebrain instead of a cortical VOI.

** indicates the distribution volume ratio (DVR), equal to the binding potential (BP_ND_) plus one. 2/3D = two/three-dimensional, EM = emission, MAP = maximum a posteriori, OSEM = ordered subset expectation maximization, FORE = Fourier rebinning, FBP = filtered back projection, o = used, x = missing, n.r. = not reported, p.i. = post injection, tg = transgenic, SC = scatter correction, AT = attenuation correction, M = motion correction, [^11^C]-PiB = [^11^C]-Pittsburgh Compound B, [^18^F]-FDDNP = 2-(1-(6-[(2-^18^F-fluoroethyl)(methyl)amino]-2-naphthyl)ethylidene)-malononitrile. PET Scanners: NIH = NIH Advanced Technology Laboratory Animal Scanner (ATLAS); F120/220 = Micro-PET Focus 120/220 Animal Scanner (Siemens Medical Solutions USA, Knoxville, TN); Inveon = Preclinical Inveon PET (Siemens Medical Solutions USA, Knoxville, TN); R4 = Concorde Microsystems microPET R4.

### 4.6 Protein quantification and plaque load

Based on our literature review, high Aß42 protein levels (> 100 ng/mg wet brain) have been found in APP23, G384A, PS2APP, ARTE10 and 5xFAD mice, even at ages less than 12 months in the latter four strains (**Table 3**). In contrast, Tg2576, APPswe, and APP/PS1dE9 mice had far less protein accumulation, even in aged animals. In animals for which plaque load measurements were also available, there was a close correspondence between histological load and Aß42 protein levels. Aß42 concentration in APP/PS1-21 mice increased from 11 ng/mg wet brain at three months of age to 67 ng/mg wet brain at eight months of age, and was not assessed at greater ages, whereas the histological plaque load in these animals further doubled at 19 months of age. Protein levels and plaque load results derive from different publications using a variety of methodologies for the assessment. Comparisons between different mouse strains should therefore be made with some caution.

### 4.7 Limitations

The findings reported for G384A and APP/PS1dE9 mice are qualified by the small sample size per time point in these strains. Further PET scans could not be obtained in these strains due to unavailability of additional mice.

## Conclusion

Present findings support the use of double transgenic PS2APP mice for early detection of cortical β-amyloid deposition by means of [^18^F]-florbetaben PET, in conjunction with PVEC. Uniform standardized procedures offer the capability for valid comparisons of β-amyloid PET results in different AD mouse strains. Interpretation of PET results in specific models is facilitated in a cross-sectional context, which removes most sources of differences between studies. The question if single or multi transgenic models are better suited for future treatment approaches likely depends on the type of intervention to be tested.

## Supporting Information

S1 Table. (DOCX)

## References

[1] Schneider LS (2013) Alzheimer disease pharmacologic treatment and treatment research. Continuum (Minneap Minn) 19: 339–357. 10.1212/01.CON.0000429180.60095.d0 23558481 PMC10564039

[2] Johnson KA, Minoshima S, Bohnen NI, Donohoe KJ, Foster NL, et al (2013) Appropriate use criteria for amyloid PET: a report of the Amyloid Imaging Task Force, the Society of Nuclear Medicine and Molecular Imaging, and the Alzheimer’s Association. J Nucl Med 54: 476–490. 10.2967/jnumed.113.120618 23359661

[3] Rominger A, Brendel M, Burgold S, Keppler K, Baumann K, et al (2013) Longitudinal assessment of cerebral beta-amyloid deposition in mice overexpressing Swedish mutant beta-amyloid precursor protein using 18F-florbetaben PET. J Nucl Med 54: 1127–1134. 10.2967/jnumed.112.114660 23729696

[4] Manook A, Yousefi BH, Willuweit A, Platzer S, Reder S, et al (2012) Small-animal PET imaging of amyloid-beta plaques with [11C]PiB and its multi-modal validation in an APP/PS1 mouse model of Alzheimer’s disease. PLoS One 7: e31310 10.1371/journal.pone.0031310 22427802 PMC3302888

[5] Kuntner C, Kesner AL, Bauer M, Kremslehner R, Wanek T, et al (2009) Limitations of small animal PET imaging with [18F]FDDNP and FDG for quantitative studies in a transgenic mouse model of Alzheimer’s disease. Mol Imaging Biol 11: 236–240. 10.1007/s11307-009-0198-z 19214638

[6] Poisnel G, Dhilly M, Moustie O, Delamare J, Abbas A, et al (2012) PET imaging with [18F]AV-45 in an APP/PS1-21 murine model of amyloid plaque deposition. Neurobiol Aging 33: 2561–2571. 10.1016/j.neurobiolaging.2011.12.024 22277262

[7] Snellman A, Lopez-Picon FR, Rokka J, Salmona M, Forloni G, et al (2013) Longitudinal amyloid imaging in mouse brain with 11C-PIB: comparison of APP23, Tg2576, and APPswe-PS1dE9 mouse models of Alzheimer disease. J Nucl Med 54: 1434–1441. 10.2967/jnumed.112.110163 23833271

[8] Rojas S, Herance JR, Gispert JD, Abad S, Torrent E, et al (2013) In vivo evaluation of amyloid deposition and brain glucose metabolism of 5XFAD mice using positron emission tomography. Neurobiol Aging 34: 1790–1798. 10.1016/j.neurobiolaging.2012.12.027 23402900

[9] Virdee K, Cumming P, Caprioli D, Jupp B, Rominger A, et al (2012) Applications of positron emission tomography in animal models of neurological and neuropsychiatric disorders. Neurosci Biobehav Rev 36: 1188–1216. 10.1016/j.neubiorev.2012.01.009 22342372

[10] Fodero-Tavoletti MT, Brockschnieder D, Villemagne VL, Martin L, Connor AR, et al (2012) In vitro characterization of [(18)F]-florbetaben, an Abeta imaging radiotracer. Nucl Med Biol 39: 1042–1048. 10.1016/j.nucmedbio.2012.03.001 22503458

[11] Zhang W, Oya S, Kung MP, Hou C, Maier DL, et al (2005) F-18 stilbenes as PET imaging agents for detecting beta-amyloid plaques in the brain. J Med Chem 48: 5980–5988. 16162001 10.1021/jm050166g PMC2593886

[12] Zhang W, Oya S, Kung MP, Hou C, Maier DL, et al (2005) F-18 Polyethyleneglycol stilbenes as PET imaging agents targeting Abeta aggregates in the brain. Nucl Med Biol 32: 799–809. 16253804 10.1016/j.nucmedbio.2005.06.001

[13] Brendel M, Delker A, Rotzer C, Boning G, Carlsen J, et al (2014) Impact of partial volume effect correction on cerebral beta-amyloid imaging in APP-Swe mice using [(18)F]-florbetaben PET. Neuroimage 84: 843–853. 10.1016/j.neuroimage.2013.09.017 24055703

[14] Richards JG, Higgins GA, Ouagazzal AM, Ozmen L, Kew JN, et al (2003) PS2APP transgenic mice, coexpressing hPS2mut and hAPPswe, show age-related cognitive deficits associated with discrete brain amyloid deposition and inflammation. J Neurosci 23: 8989–9003. 14523101 10.1523/JNEUROSCI.23-26-08989.2003 PMC6740398

[15] Ozmen L, Albientz A, Czech C, Jacobsen H (2009) Expression of transgenic APP mRNA is the key determinant for beta-amyloid deposition in PS2APP transgenic mice. Neurodegener Dis 6: 29–36. 10.1159/000170884 19066434

[16] Busche MA, Eichhoff G, Adelsberger H, Abramowski D, Wiederhold KH, et al (2008) Clusters of hyperactive neurons near amyloid plaques in a mouse model of Alzheimer’s disease. Science 321: 1686–1689. 10.1126/science.1162844 18802001

[17] Jankowsky JL, Xu G, Fromholt D, Gonzales V, Borchelt DR (2003) Environmental enrichment exacerbates amyloid plaque formation in a transgenic mouse model of Alzheimer disease. J Neuropathol Exp Neurol 62: 1220–1227. 14692698 10.1093/jnen/62.12.1220

[18] Xiong H, Callaghan D, Wodzinska J, Xu J, Premyslova M, et al (2011) Biochemical and behavioral characterization of the double transgenic mouse model (APPswe/PS1dE9) of Alzheimer’s disease. Neurosci Bull 27: 221–232. 10.1007/s12264-011-1015-7 21788993 PMC5560305

[19] Rousset OG, Collins DL, Rahmim A, Wong DF (2008) Design and implementation of an automated partial volume correction in PET: application to dopamine receptor quantification in the normal human striatum. J Nucl Med 49: 1097–1106. 10.2967/jnumed.107.048330 18552147 PMC3104499

[20] Rousset OG, Ma Y, Evans AC (1998) Correction for partial volume effects in PET: principle and validation. J Nucl Med 39: 904–911. 9591599

[21] Klunk WE, Bacskai BJ, Mathis CA, Kajdasz ST, McLellan ME, et al (2002) Imaging Abeta plaques in living transgenic mice with multiphoton microscopy and methoxy-X04, a systemically administered Congo red derivative. J Neuropathol Exp Neurol 61: 797–805. 12230326 10.1093/jnen/61.9.797

[22] Radde R, Bolmont T, Kaeser SA, Coomaraswamy J, Lindau D, et al (2006) Abeta42-driven cerebral amyloidosis in transgenic mice reveals early and robust pathology. EMBO Rep 7: 940–946. 16906128 10.1038/sj.embor.7400784 PMC1559665

[23] Guntert A, Dobeli H, Bohrmann B (2006) High sensitivity analysis of amyloid-beta peptide composition in amyloid deposits from human and PS2APP mouse brain. Neuroscience 143: 461–475. 17008022 10.1016/j.neuroscience.2006.08.027

[24] Maeda J, Zhang MR, Okauchi T, Ji B, Ono M, et al (2011) In vivo positron emission tomographic imaging of glial responses to amyloid-beta and tau pathologies in mouse models of Alzheimer’s disease and related disorders. J Neurosci 31: 4720–4730. 10.1523/JNEUROSCI.3076-10.2011 21430171 PMC3251921

[25] Maeda J, Ji B, Irie T, Tomiyama T, Maruyama M, et al (2007) Longitudinal, quantitative assessment of amyloid, neuroinflammation, and anti-amyloid treatment in a living mouse model of Alzheimer’s disease enabled by positron emission tomography. J Neurosci 27: 10957–10968. 17928437 10.1523/JNEUROSCI.0673-07.2007 PMC6672864

[26] Garcia-Alloza M, Robbins EM, Zhang-Nunes SX, Purcell SM, Betensky RA, et al (2006) Characterization of amyloid deposition in the APPswe/PS1dE9 mouse model of Alzheimer disease. Neurobiol Dis 24: 516–524. 17029828 10.1016/j.nbd.2006.08.017

[27] Savonenko A, Xu GM, Melnikova T, Morton JL, Gonzales V, et al (2005) Episodic-like memory deficits in the APPswe/PS1dE9 mouse model of Alzheimer’s disease: relationships to beta-amyloid deposition and neurotransmitter abnormalities. Neurobiol Dis 18: 602–617. 15755686 10.1016/j.nbd.2004.10.022

[28] Horgan J, Miguel-Hidalgo JJ, Thrasher M, Bissette G (2007) Longitudinal brain corticotropin releasing factor and somatostatin in a transgenic mouse (TG2576) model of Alzheimer’s disease. J Alzheimers Dis 12: 115–127. 17917156 10.3233/jad-2007-12201 PMC2919580

[29] Hsiao K, Chapman P, Nilsen S, Eckman C, Harigaya Y, et al (1996) Correlative memory deficits, Abeta elevation, and amyloid plaques in transgenic mice. Science 274: 99–102. 8810256 10.1126/science.274.5284.99

[30] Kawarabayashi T, Younkin LH, Saido TC, Shoji M, Ashe KH, et al (2001) Age-dependent changes in brain, CSF, and plasma amyloid (beta) protein in the Tg2576 transgenic mouse model of Alzheimer’s disease. J Neurosci 21: 372–381. 11160418 10.1523/JNEUROSCI.21-02-00372.2001 PMC6763819

[31] Oakley H, Cole SL, Logan S, Maus E, Shao P, et al (2006) Intraneuronal beta-amyloid aggregates, neurodegeneration, and neuron loss in transgenic mice with five familial Alzheimer’s disease mutations: potential factors in amyloid plaque formation. J Neurosci 26: 10129–10140. 17021169 10.1523/JNEUROSCI.1202-06.2006 PMC6674618

[32] Beckmann N, Gerard C, Abramowski D, Cannet C, Staufenbiel M (2011) Noninvasive magnetic resonance imaging detection of cerebral amyloid angiopathy-related microvascular alterations using superparamagnetic iron oxide particles in APP transgenic mouse models of Alzheimer’s disease: application to passive Abeta immunotherapy. J Neurosci 31: 1023–1031. 10.1523/JNEUROSCI.4936-10.2011 21248127 PMC6632947

[33] Bondolfi L, Calhoun M, Ermini F, Kuhn HG, Wiederhold KH, et al (2002) Amyloid-associated neuron loss and gliogenesis in the neocortex of amyloid precursor protein transgenic mice. J Neurosci 22: 515–522. 11784797 10.1523/JNEUROSCI.22-02-00515.2002 PMC6758656

[34] Kuo YM, Beach TG, Sue LI, Scott S, Layne KJ, et al (2001) The evolution of A beta peptide burden in the APP23 transgenic mice: implications for A beta deposition in Alzheimer disease. Mol Med 7: 609–618. 11778650 PMC1950067

[35] Sturchler-Pierrat C, Abramowski D, Duke M, Wiederhold KH, Mistl C, et al (1997) Two amyloid precursor protein transgenic mouse models with Alzheimer disease-like pathology. Proc Natl Acad Sci U S A 94: 13287–13292. 9371838 10.1073/pnas.94.24.13287 PMC24301

[36] Willuweit A, Velden J, Godemann R, Manook A, Jetzek F, et al (2009) Early-onset and robust amyloid pathology in a new homozygous mouse model of Alzheimer’s disease. PLoS One 4: e7931 10.1371/journal.pone.0007931 19936202 PMC2775952

[37] Toyama H, Ye D, Ichise M, Liow JS, Cai L, et al (2005) PET imaging of brain with the beta-amyloid probe, [11C]6-OH-BTA-1, in a transgenic mouse model of Alzheimer’s disease. Eur J Nucl Med Mol Imaging 32: 593–600. 15791432 10.1007/s00259-005-1780-5

[38] von Reutern B, Grunecker B, Yousefi BH, Henriksen G, Czisch M, et al (2013) Voxel-based analysis of amyloid-burden measured with [(11)C]PiB PET in a double transgenic mouse model of Alzheimer’s disease. Mol Imaging Biol 15: 576–584. 10.1007/s11307-013-0625-z 23572425

[39] Dorr A, Sled JG, Kabani N (2007) Three-dimensional cerebral vasculature of the CBA mouse brain: a magnetic resonance imaging and micro computed tomography study. Neuroimage 35: 1409–1423. 17369055 10.1016/j.neuroimage.2006.12.040

