# Supplementary material for: Cross-Sectional Comparison of Small Animal [18F]-Florbetaben Amyloid-PET between Transgenic AD Mouse Models
Source: PLoS One. 2015 Feb 23;10(2):e0116678. doi: 10.1371/journal.pone.0116678 (PMC4338066; doi:10.1371/journal.pone.0116678)
Supplement: S1 Table. — (DOCX) [file pone.0116678.s001.docx]

***Supplemental Table S1***

| **Strain, Age and Animal Numbers** | **Methoxy-X04** | **Plaque Size**  **%-Distribution** |
| --- | --- | --- |
| PS2APP  8mo  (N = 1) | 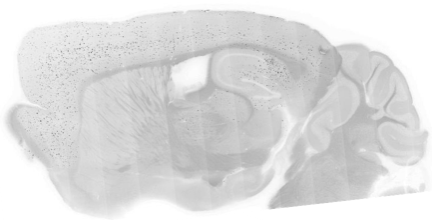 | 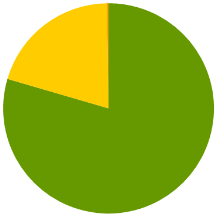 |
| PS2APP  12mo  (N = 2) | 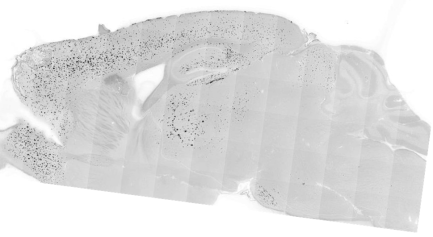 | 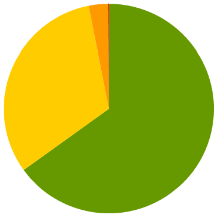 |
| PS2APP  19mo  (N = 2) | 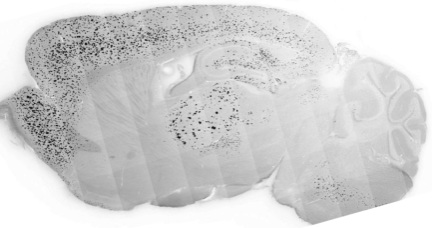 | 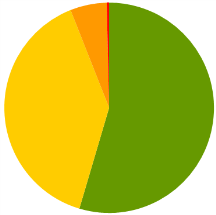 |
| G384A  5.5mo  (N = 2) | 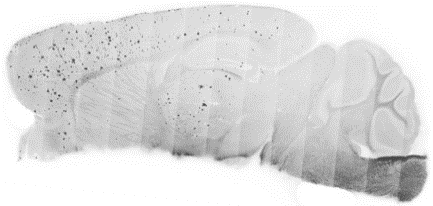 | 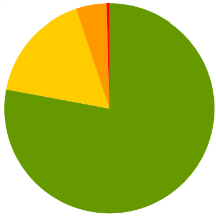 |
| G384A  16mo  (N = 1) | 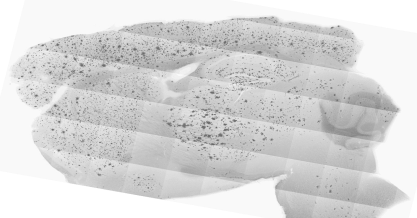 | 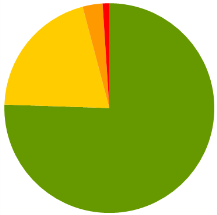 |
| APP/PS1dE9  12mo  (N = 2) | 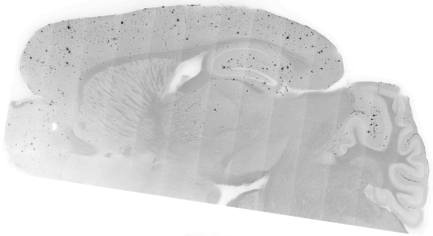 | 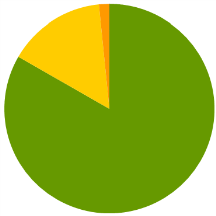 |
| APP/PS1dE9  24mo  (N = 2) | 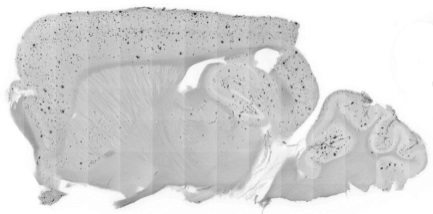 | 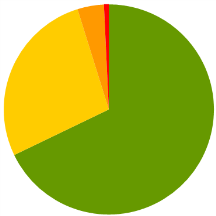 |
| APPswe  20mo  (N = 5) | 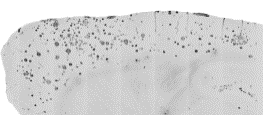* | 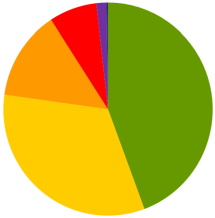 |
|  |  | 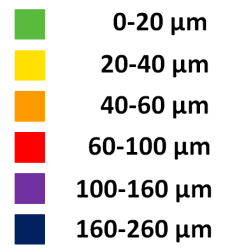 |

***Supplemental Table S1 Caption***

Illustrative comparison of histological results obtained from the four different AD mouse strains (column 1). Sagittal slices (column 2) show methoxy-X04 staining of ß-amyloid plaques. The distribution of plaque sizes is visualized in column 3. *For APPswe mice, only the frontal cortex was imaged.
